# Decoupling behavioral and transcriptional responses to color in an eyeless cnidarian

**DOI:** 10.1186/s12864-020-6766-y

**Published:** 2020-05-14

**Authors:** Whitney B. Leach, Adam M. Reitzel

**Affiliations:** Department of Biological Sciences, University of North Carolina at Charlotte, 9201 University City Blvd, Woodward Hall, Room 381A, Charlotte, NC 28223 USA

**Keywords:** *Nematostella*, Behavior, Gene expression, Transcriptomics, Photoperiod

## Abstract

**Background:**

Animals have specific molecular, physiological, and behavioral responses to light that are influenced by wavelength and intensity. Predictable environmental changes – predominantly solar and lunar cycles – drive endogenous daily oscillations by setting internal pacemakers, otherwise known as the circadian clock. Cnidarians have been a focal group to discern the evolution of light responsiveness due to their phylogenetic position as a sister phylum to bilaterians and broad range of light-responsive behaviors and physiology. Marine species that occupy a range of depths will experience different ranges of wavelengths and light intensities, which may result in variable phenotypic responses. Here, we utilize the eyeless sea anemone *Nematostella vectensis*, an estuarine anemone that typically resides in shallow water habitats, to compare behavioral and molecular responses when exposed to different light conditions.

**Results:**

Quantitative measures of locomotion clearly showed that this species responds to light in the blue and green spectral range with a circadian activity profile, in contrast to a circatidal activity profile in the red spectral range and in constant darkness. Differences in average day/night locomotion was significant in each condition, with overall peak activity during the dark period. Comparative analyses of 96 transcriptomes from individuals sampled every 4 h in each lighting treatment revealed complex differences in gene expression between colors, including in many of the genes likely involved in the cnidarian circadian clock. Transcriptional profiling showed the majority of genes are differentially expressed when comparing mid-day with mid-night, and mostly in red light. Gene expression profiles were largely unique in each color, although animals in blue and green were overall more similar to each other than to red light.

**Conclusions:**

Together, these analyses support the hypothesis that cnidarians are sensitive to red light, and this perception results in a rich transcriptional and divergent behavioral response. Future work determining the specific molecular mechanisms driving the circadian and potential circatidal rhythms measured here would be impactful to connect gene expression variation with behavioral variation in this eyeless species.

## Background

Light can be a rich source of environmental information depending on an organism’s ability to detect it, otherwise known as ‘photoreception’. Light intensity and duration are indicative of the time of day and season, respectively, which provides a central signal for regulating behavior and physiology [1–3]. The particular wavelengths that compose visible light represent complex information; for example, light attenuation in water, where longer wavelengths are absorbed more quickly over depth, provides a signal for position in the water column. The spectral composition of light also varies depending on the relative position of the sun such that light quality is indicative of time of day [4]. Spectral irradiance from moonlight is also a source of information that varies in intensity dependent on the phase of the moon [5]. Further, reflected light from the moon is a widely utilized cue for regulating the behavior, physiology, and reproductive cycle of many animals [1, 6–8].

Cnidarians have been a critical taxonomic lineage for understanding the evolution of photoreception in animals and the impacts of light on behavior and physiology. Most cnidarians lack specialized visual structures and thus the reception and transduction of light signals is performed extraocularly (outside the eyes), primarily with two photopigment types: cryptochromes and opsins [9]. Light detection is important for eyeless species, where dedicated visual organs are not present, and a strong pressure for environmental entrainment still exists. In cnidarian species, light has been shown to be a central entraining cue for a broad range of behavioral and molecular responses [10–12] and is the predicted primary cue for circadian entrainment [13, 14]. For example, the diel (daily) vertical migrations of jellyfish are timed to daily light oscillations [15–17], and the reproduction of many reef building corals is entrained to lunar moonlight cycles [18], (but see [19]), which correlates with expression of cryptochromes [20]. Moreover, individual wavelengths of light and portions of the light spectrum have been shown to result in specific behaviors, including larval settlement [21, 22], adult activity [23], and cnidocyte (stinging cells) discharge [24]. Opsins have also been identified with tissue restricted expression in the gonads [25], oral region, and tentacles [26] of certain species, which may be associated with specific physiological processes. In many cases, the display of diel behaviors are attributed to an endogenous time keeping mechanism or ‘circadian clock’ (~ 24-h rhythms) in cnidarians [13].

The connections between light-dependent behaviors and molecular responses remain poorly understood in any cnidarian, particularly for species with only extraocular photoreception, and even less understood are the mechanisms for entrainment to additional environmental factors like temperature, oxygen, nutrients or tidal patterns. Previous research with the estuarine sea anemone *Nematostella vectensis* (the focus of this study; hereafter, *Nematostella*) has shown that light exposure impacts reproduction [27], respiration [28], and locomotion [29], similar to other cnidarians. In addition to circadian behavior, many intertidal organisms often exhibit circatidal or “twice-daily” behaviors [30]; however, only one study has reported these behavioral patterns in *Nematostella* despite its estuarine habitat [29].

Here, we utilize a combinatory organismal and molecular approach testing two hypotheses regarding light entrainment in *Nematostella.* First, we hypothesized that in response to diel red, green and blue light, *Nematostella* would exhibit higher activity at night (during the ‘scotoperiod’) and lower activity during the day (the ‘photoperiod), mimicking behavior under full spectrum ‘white’ light used in earlier studies. Second, exposure to different colors of light would result in observable behavioral shifts that correlate to transcriptional remodeling of circadian clock-related genes (e.g., *Clock, PAR-bZIPs*) or differential expression of light-responsive genes (e.g., cryptochromes, opsins). We quantitatively measured the behavior of sea anemones exposed to light:dark (12:12) cycles of either 1) red light; 2) green light; 3) blue light; or constant darkness, as well as qualitatively monitored female reproductive output. We used tag-based RNA-sequencing to transcriptionally profile animals from each light condition and compared gene expression profiles from each color and over time. Comparing between light treatments allowed us to identify expression patterns that might indicate a narrow range of light sensitivity and comparisons between time points in each light treatment further provided an opportunity to look for time-of-day dependent molecular responses. Together, our results show that *Nematostella* is capable of photo-entrainment in each diel condition evident by behavioral cycles and reproductive output and further exhibit differential behavioral and molecular profiles conditional to light color which points to red light sensitivity in this species.

## Results

### Nocturnal behavior irrespective of light condition

**Fig. 1:**
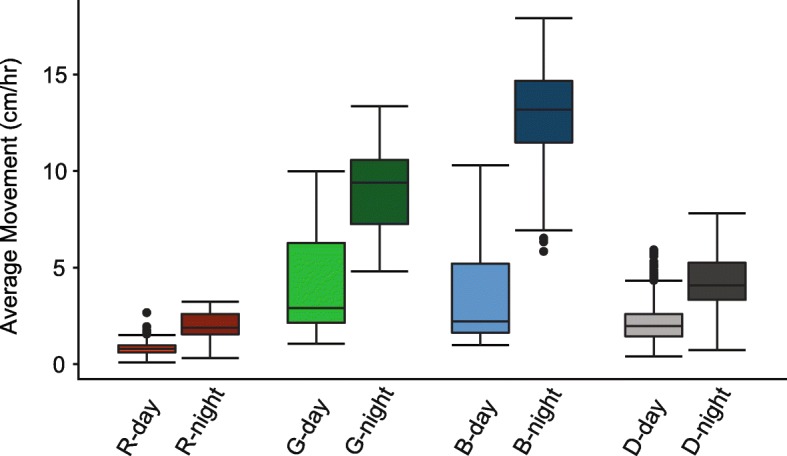
Average locomotive activity (cm/hr) of *Nematostella vectensis* during the photoperiod (light bars, left) versus scotoperiod (dark bars, right) over 48 h in each light condition (R – red, G – green B – blue, D – dark). All comparisons between photoperiod and scotoperiod of each color and within colors were significant (two-way ANOVA with Tukey post hoc tests). All statistical values for pairwise comparisons can be found in Table S1

We monitored activity using animal tracking software (see Methods), in which each sea anemone was measured individually after entrainment in red, green, or blue light:dark (LD) conditions (here after referred to as ‘X color light’) or dark:dark (DD) conditions for 48 continuous hours. Plotting locomotion (total distance moved) over time for all treatments (red light, green light, blue light, and DD) revealed that the average activity of sea anemones was significantly higher during the scotoperiod than during the photoperiod for each condition (Fig. 1). Locomotion increased from red to green to blue light, with animals in blue light displaying the highest overall activity (Fig. 1). Average movement in both the photo- and scotoperiod was significantly different within and between treatments (Table S1).

### Light color-inducible circadian or circatidal behavioral response

**Fig. 2:**
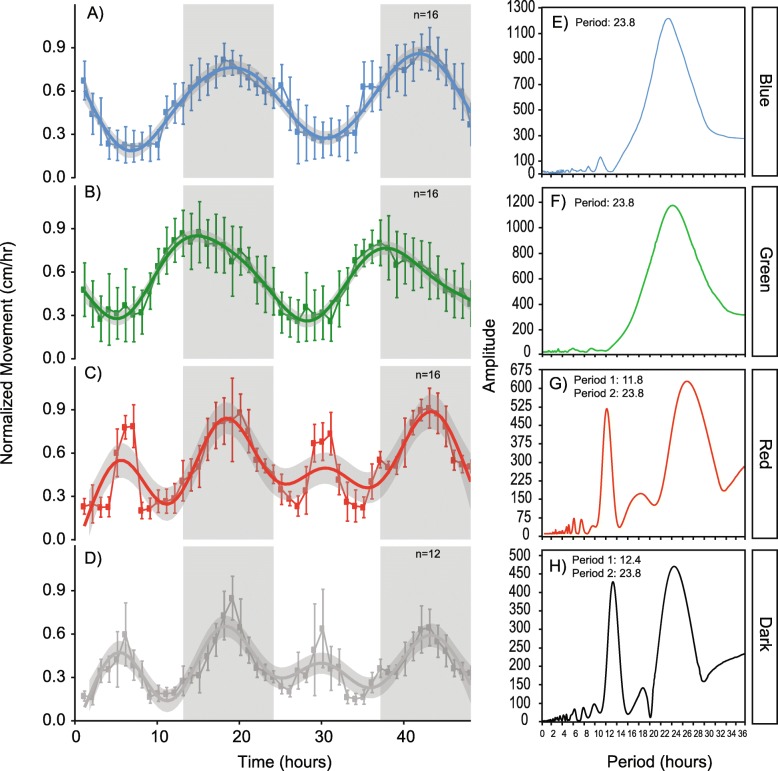
Normalized locomotive activity patterns of *Nematostella vectensis* over time (left panel) in **a** blue light:dark conditions, **b** green light:dark conditions, **c** red light:dark conditions, and **d** constant dark:dark conditions. The 48-h time course is indicated by the x-axis, and normalized movement (cm/hr) on the y-axis of the left panel behavioral plots. White and grey boxes in the plot area indicate the light:dark cycle, or the photoperiod and scotoperiod of the time course, respectively. Each data point on the behavioral plots represent *n* replicates (*n*blue = 16; *n*green = 16, *n*red = 16; *n*dark = 12). The right panel (**e**-**h**) shows periodograms corresponding to each color (annotated in the far-right box) using Chi-square analysis from activity data for *n* individuals in each light condition (confidence interval < 0.01). Periodicity values are reported in the top left corner of each graph

Sea anemones in blue and green light exhibited locomotor oscillations that parallel circadian behavior of animals under full spectrum diel conditions. This response was signified by nocturnal movement, with peak activity occurring during the scotoperiod (Fig. 2a, b). Animals in red light and in DD displayed behavioral patterns consistent with circatidal oscillations, or ‘twice-daily’ rhythms. In addition to nocturnal peaks of activity during the scotoperiod, a second peak during the photoperiod was observed in red light and in DD (Fig. 2c, d). We used chi-squared analysis to determine the periodicity of animals from each light treatment using a confidence level of 0.01. Over the 48-h time course, animals entrained to blue and green light had a periodicity of 23.8-h (Fig. 2e, f). A periodicity of 11.8-h and 12.4-h was observed for red light and DD, respectively (Fig. 2g, h).

### Color insensitive gametogenesis of *Nematostella*

Groups of adult female sea anemones were induced to spawn in red, green, and blue light conditions, otherwise following reliable spawning procedures determined by Fritzenwanker & Technau [31]. Qualitative measurements of gametogenesis (i.e., egg output) from each group were recorded weekly and showed sea anemone spawning occurred in all colors of light.

### Light color and time-of-day dependent transcriptional response

**Fig. 3:**
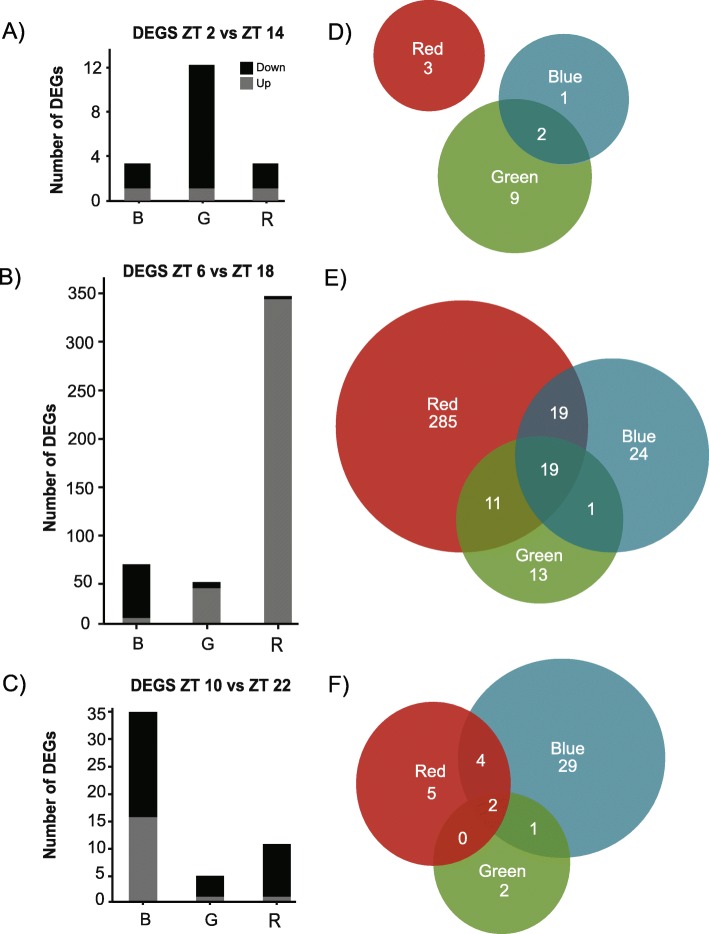
Time-of-day and light-dependent differential gene expression analysis of *Nematostella vectensis.***a**-**c** Counts of differentially expressed (DE) genes between the day (ZT = 2, 6, 10) and night (ZT = 14, 18, 22) timepoints in each light treatment (B – blue, G – green, R – red). Up- and down-regulated genes with respect to the photoperiod are shown with black (down-regulated) and grey (up-regulated) bars (i.e., if up-regulated, genes are up during the photoperiod compared to the scotoperiod). **d**-**f** Venn diagrams of DE genes shared between each light condition. No genes were differently expressed between the different color light conditions and constant dark conditions; thus, they are not represented in this figure

To identify genes differentially expressed in each light treatment, we sequenced transcriptomes of 96 sea anemones: 24 individuals per condition sampled in 4-h intervals over the period of 1 day. Four replicates per time point were prepared for sequencing from each treatment. Tag-based RNA sequencing produced > 225 million reads (Table S2). On average, there were 2.3 million 100-base single-end reads per sample. Using a standard bioinformatic processing pipeline, reads were quality filtered and PCR duplicates were removed, leaving an average of 621,629 reads per sample (Tables S2 [32, 33];). After trimming, reads were mapped to the Vienna *Nematostella* transcriptome (~ 24,000 genes) with an average mapping efficiency of 75.17%. Using DESeq2, raw count data were filtered, transformed, and normalized prior to statistical analysis employing Wald tests. Over the 24-h sampling period of all light treatments, 512 transcripts were identified to have diel patterns of expression passing a Benjamini-Hochberg FDR cutoff of 10%. Of these diel genes, both light color (red, green, blue) and the time-of-day were contributing factors for their expression. Of the 512 genes, 441 (86%) were differentially expressed between Zeitgeber time (ZT; term used to describe time defined by lights on at ZT = 0) = 6 vs. ZT = 18 (mid-day and mid-night contrast); 18 genes differentially expressed between ZT = 2 vs. ZT = 14 (early photoperiod and early scotoperiod contrast); and 52 genes were differentially expressed between ZT = 10 vs. ZT = 22 (late photoperiod and late scotoperiod contrast) (Fig. 3d-e). Of the 512 time-of-day dependent diel genes, 348 (68%) were differentially expressed in red light; 102 (20%) were differentially expressed in blue light, 60 (12%) were differentially expressed in green light; and two were differentially expressed in dark conditions, with minimal overlap between light treatments. A list of differentially expressed genes from each comparison can be found in Table 1 and Table S3.

**Table 1:** Genes of interest and associated Vienna transcriptome IDs

DEGs of Interest	
ID	Annotation
NVE2080	*Clock*
NVE1138	*Cry1a*
NVE24214	*Cry1b*
NVE14677	*PAR-bZIPa*
NVE20636	*PAR-bZIPb*
NVE8107	*PAR-bZIPc*
NVE8085	*PAR-bZIPd*
NVE8679	*helt*
NVE4116	*CiPC*
NVE19057	*Timeout*
NVE4209	*HSP70A*
NVE15435	*HSP70B*
NVE2172	*HSP70E*

The majority of diel genes from the mid-day and mid-night contrast, 419 out of 441 (95%), were up-regulated during the photoperiod (Fig. 3b). More than two thirds of these diel genes were unique to red light (285 out of 441, 68%), and less than 1 % of these genes were down-regulated during the photoperiod (22 out of 441). Notably, the *timeless* homolog, *nvTimeout*, was uniquely differentially expressed under red light and was 1.5-fold higher during the photoperiod. Further, several heat shock proteins were up-regulated mid-day in red light only (i.e., *nvHSP70E*, *nvHSP90A*, and *nvHSP90B*). Twenty-four out of 441 diel genes were unique to blue light, 75% of which were upregulated during the photoperiod, and included the circadian clock candidate genes *nvPAR-bZIPa*, *nvPAR-bZIPd*, and *nvhelt.* The transcription factor *nvPAR-bZIPc* was one of eight genes down-regulated during the photoperiod of blue light, and decreased > 2.5-fold after the light-dark transition, consistent with findings from Leach and Reitzel [33] and Reitzel et al. [13]. Diel genes with the strongest changes in expression under blue light were core histone proteins, with a > 7-fold increase during the photoperiod. Few diel genes (13 out of 441) were uniquely expressed in green light and 53% were upregulated during the photoperiod (7 genes). Only one gene, *supervillin*, was differentially expressed in DD, and was > 3-fold higher mid-day. A small proportion of diel genes were shared between all light treatments (19 out of 441; Fig. 3), and primarily consisted of cytoskeletal proteins (i.e., *alpha*-*tubulin*, *supervillin*). Nineteen diel genes (of 441) were shared between red light and blue light, including *nvPAR-bZIPa,* a previously characterized diurnal gene [13]*.* In both conditions, *nvPAR-bZIPa* transcription was > 2-fold greater during the photoperiod.

Although more than 85% of all diel genes were differentially expressed between mid-day and mid-night, a portion of diel genes (18 out of 512; ~3%) were only identified in the contrast between early morning and early night following the light-dark transition. Of these 18 diel genes, 83% were up-regulated during the photoperiod (Fig. 3a). Three diel genes were uniquely expressed in red light: a perilipin-like protein, a selenoprotein precursor, and an unannotated gene. One diel gene, a protease inhibitor, was uniquely expressed in blue light. Nine diel genes were uniquely expressed in green light and primarily consisted of unannotated proteins. No genes were shared between all treatments, between red light/blue light or between red light/green light (Fig. 3d); however, two genes, both PAR-bZIP transcription factors, were shared between blue light and green light: *nvPAR-bZIPa* and another PAR-bZIP with high sequence similarity to *nvPAR-bZIPa*, up-regulated during the photoperiod of each light condition, as was also observed in the mid-day and mid-night comparison. One gene was differentially expressed in constant darkness and was unannotated (Table S3).

Fifty-two diel genes were differentially expressed between late day and late night; the time point just prior to the transitions in lighting. Of these, < 35% were up-regulated during the photoperiod (Fig. 3c). Unique to blue light was the transcription factor *nvPAR-bZIPd* and a heat shock protein *nvHSP70C*, both up-regulated during the photoperiod (Table S3). Two genes were shared between all conditions, with the same directionality of expression: up during the scotoperiod (an unannotated gene and *collagenase*). One gene, *ester hydrolase*, was shared between blue light and green light and four genes were shared between red light and blue light (2 unannotated genes, *carboxypeptidase*, and a ribosomal protein; Table S3). There were no genes in DD that were differentially expressed late in the light cycle.

Gene ontology (GO) enrichment analysis of genes differentially expressed in response to individual colors revealed that under red light, down-regulated genes enriched in the biological process category were related to ‘G-protein coupled receptor signaling’ and ‘regulation of response to stress’. A survey of 31 candidate opsin genes (members of the G-protein coupled receptors superfamily) identified by Suga et al. [26], revealed no color or time-dependent differential expression. Conversely, up-regulated molecular function genes were enriched for ‘mRNA metabolic process’ and ‘methylation’ in red light. GO enrichment analysis discovered in both red light and green light genes relating to ‘activation of immune response’ were down-regulated, and ‘cellular respiration’ genes were up-regulated. Enriched terms in blue light included up-regulated genes involved ‘DNA binding’, ‘chromatin binding’, and ‘amide biosynthetic process’, and down-regulated genes in the ‘signaling receptor binding’ category. There was shared enrichment amongst up-regulated genes of the GO terms ‘biological phase’ and ‘oxidation-reduction’ for each color treatment.

### Transcriptomic response combining light color and time

Weighted gene co-expression networks were constructed using 4965 filtered genes (see Methods) to classify systems-level molecular responses to different light colors. Each gene in the data set was assigned to an expression module, pairing them based on similarity of expression profiles using a weighted gene correlation network and given an arbitrary color name (not to be confused with the light colors). In total, 10 co-expression modules resulted from the analysis, and eight were highly enriched for genes corresponding to specific light colors (Figure S3). Three module eigengenes were composed of enriched genes negatively associated, or down-regulated, with blue light (greenyellow: − 0.27, *p* < 0.009; lightcyan: − 0.36, *p* < 0.0004; purple: − 0.36, *p* < 0.0005), while one eigengene was positively associated, or up-regulated, with blue light (grey60: 0.32, *p* < 0.002). The strongest module negatively associated with blue light (purple) exhibited GO enrichment of ‘receptor regulator activity’ and ‘activation of immune response’. GO analysis of genes from the module eigengene positively associated with blue light (grey60) did not find any enriched terms. The co-expression network returned two modules that were enriched for genes specific to green light conditions, both of which were up-regulated (greenyellow: 0.26, *p* < 0.01; pink: 0.3, *p* < 0.004). GO analysis of green light specific modules identified functional enrichment of the terms ‘cation binding’ and ‘immune system development’. Two modules containing genes enriched for red light conditions were identified, and each of these were up-regulated in response to red light (turquoise: 0.31, *p* < 0.002; lightcyan: 0.22, *p* < 0.04); however, expression of the turquoise module eigengene was down-regulated in DD conditions and the lightcyan module contained genes that were down-regulated in blue light and up-regulated in DD. The GO terms ‘DNA-binding transcription factor activity’, ‘signaling receptor activity’ and ‘molecular transducer activity’ were positively enriched in response to red light. Several modules were positively associated with DD (midnightblue: 0.29, *p* < 0.006; black: 0.37, *p* < 0.0003; lightcyan: 0.23, *p* < 0.02; purple: 0.43, *p* < 0.00002) and negatively associated with DD (grey60: − 0.21, *p* < 0.05; pink: − 0.22, *p* < 0.03; turquoise: − 0.29, *p* < 0.004). The purple and black modules were most strongly up-regulated in DD and were enriched for GO terms related to ‘activation of immune response’ and ‘antioxidant/peroxidase activity’, respectively. A list of all modules is provided in Table S4.

Although some modules were not positively or negatively associated with a specific light treatment, the co-expression network identified modules that were associated with a specific time point during the day (Table S4). The blue module contained genes that were up-regulated during the photoperiod (ZT = 6, 0.28, *p* < 0.006), and down-regulated the scotoperiod (ZT = 14, − 0.21, *p* < 0.04). GO analysis of this module found the terms ‘NADH dehydrogenase activity’ and ‘cellular respiration’ to be enriched at specific points of the day, consistent with previous respirometry data [28]. The salmon module was not enriched for specific GO terms, however genes in this module were downregulated during the scotoperiod (ZT = 18, − 0.27, *p* < 0.009).

### Expression of candidate circadian genes

**Fig. 4:**
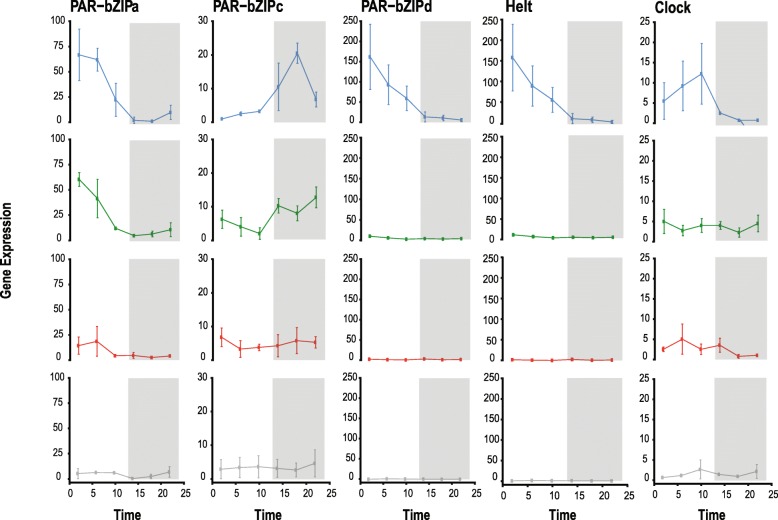
*Nematostella vectensis* candidate circadian gene profiles over the 24-h sequencing time course, organized by gene and color. Each vertical set of plots represents a single gene’s expression in each light condition in the following order: blue, green, red, dark. Data points on each plot represent four individually sequenced animals. Error bars are calculated from the standard error of the mean for each data point (*n* = 4). The time course of the experiment is shown along the x-axis, and the normalized expression values are shown along the y-axis. Note the scale is the same for each light condition of a specific gene, but the scales differ across genes. White and grey boxes in the plot area indicate the light:dark cycle, or the photoperiod and scotoperiod of the time course, respectively. All statistical values for comparisons can be found in Table S5

**Fig. 5:**
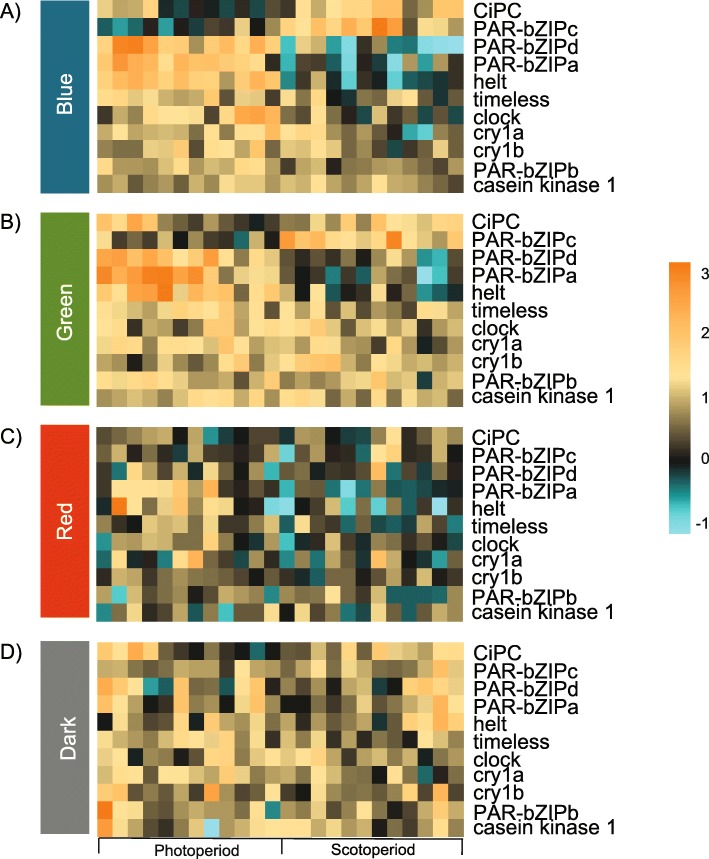
Heatmap of *Nematostella vectensis* candidate circadian genes across each light treatment **a** blue, **b** green, **c** red, **d** dark. The experiment key at the bottom of the lower heatmap identifies the ‘photoperiod’ and ‘scotoperiod’ sampling points of the 24-h time course. The columns represent each individual animal that was sampled during the experiment. Three time points were collected during the photoperiod and three time points were collected during the scotoperiod for a total of six collection time points. At each time point, four replicate animals were collected (24 total animals sampled in each treatment; 4 treatments × 6 time points = 96 total animals). Each row of the heatmaps shows expression of a single annotated gene, labelled on the right. The color scale is log2 fold change

Diel patterns of expression for genes previously described as circadian were observed differently across light conditions. Transcription of *nvClock* was highest during the late photoperiod (ZT = 10) of blue light and decreased immediately following the light to dark transition, consistent with previous studies [33–35] (Figs. 4; 5a). Diel expression of *nvClock* was not observed in anemones cultured in DD, and was significantly different from blue light at each sampling point during subjective day. In green light and red light, *nvClock* expression was not significantly different from DD at any time point, but were both significantly different from blue light at ZT = 10 (*p* < 0.0001). At ZT = 6, *nvClock* expression was also significantly different between blue light and green light (*p* < 0.0001; Figs. 4, 5a-b, Table S5). *nvPAR-bZIPa* expression was highest early in the photoperiod of each color: at ZT = 2 of blue light and green light, and ZT = 6 of red light (Fig. 5c). At ZT = 2, *nvPAR-bZIPa* expression was significantly different from dark conditions in both blue light and green light, but not red light (Fig. 5). Further, at ZT = 6 each color was significantly different from all others (Table S5) excluding red light vs. dark. Transcription began decreasing during the late photoperiod and reached an expression trough just after the start of the scotoperiod of each color (ZT = 14). Transcription increased as the dark to light transition occurred (Fig. 4). This pattern was not observed under DD (Fig. 5d). Similarly, transcription of *nvPAR-bZIPd* peaked during the early photoperiod (ZT = 2) and decreased steadily into the scotoperiod. Diel expression of *nvPAR-bZIPd* was only present in blue light, but transcription in both red and green light was significantly different than blue light at ZT = 6 (*p* < 0.0001) and no differential expression was measured in DD. Conversely, *nvPAR-bZIPc* peak expression occurred during mid- and late-scotoperiod of blue light (ZT = 18) and green light (ZT = 22), respectively; however, transcription was not sustained during late subjective night into the photoperiod. While *nvPAR-bZIPc* expression was diel in blue and green light, expression was constant in red light and dark conditions. Similarly, a Hes/Hey-like gene, *nvhelt,* was diurnal only under blue light and green light, however transcription was much higher in blue light overall (Fig. 4). *nvhelt* transcription was highest at the beginning of the photoperiod (ZT = 2) and decreased over subjective day to a trough at ZT = 22. Expression of the cryptochromes *nvCry1a* and *nvCry1b* was rhythmic with peaks at mid-photoperiod (ZT = 6) of blue and green light, however significant oscillations of these transcripts were not measured under red light or dark conditions. Diurnal expression of the *circadian interacting pacemaker protein*, *nvCiPC*, was only observed under blue light (Fig. 5, Table S5). As previously shown by Reitzel et al. [34] cyclic expression of *nvCycle* and *nvCry2* was not observed.

## Discussion

**Fig. 6:**
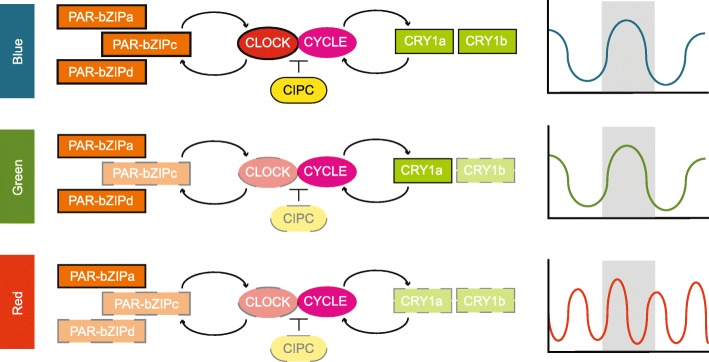
Proposed model for the cnidarian circadian clock composed of two loops adapted from Reitzel et al. [13] and associated behavior. This proposed network combines the positive elements (center), the feed-forward loop (left), and the feedback loop (right). The positive elements, *Clock* and *Cycle* heterodimerize (CLOCK:CYCLE) and upregulate genes in the feed-forward and feedback loops (PAR-bZIPs and cryptochromes, respectively) where they act as transcriptional regulators for the positive elements. CIPC is a predicted repressor of the CLOCK:CYCLE protein complex based on data from vertebrates. Differential expression (DE) of genes in this network are indicated with solid (DE) or dashed (not DE) lines for each color (key far left). *Cycle* is not outlined because this gene was not differentially expressed in any condition. Plots of behavioral responses to each wavelength are shown on the far right as cartoons

Diel light cycles synchronize predictable patterns of behavior, physiology and gene expression, generating rhythmicity via two general processes: a direct response to light or through modulation by a molecular mechanism (i.e., a circadian clock). Broadly, the molecular basis for animal circadian clocks involves interlocked transcription-translation feedback loops with positive and negative elements [36]. Through a combination of phylogenomics and light-dependent gene expression assays, Reitzel et al. [13] proposed a model for the cnidarian circadian clock composed of two loops (Fig. 6): the feedback loop where light dependent cryptochromes are involved in negative regulation of the CLOCK:CYCLE dimer and a feedforward loop where PAR-bZIPs activate and repress transcription of *Clock* or *Cycle* – as in *Drosophila* (see Cyran et al. [37]). Our transcriptome results revealed progressive loss of gene expression differences for these proposed clock components from blue to green to red light. In blue light, components of each loop are differentially expressed in light:dark conditions suggesting robust diel gene expression for all genes, some of which have different phases (e.g., *nvPAR-bZIPa* and *nvPAR-bZIPc*). In green light, individual genes in each component (i.e., *nvPAR-bZIPc*, *nvClock*, *nvCry1a)* no longer showed significant differences in expression; however, anemones maintained rhythmic activity with 24-h periodicity. In red light, only *nvPAR-bZIPa* maintained differential gene expression in light:dark, which was restricted to just a narrow time comparison (early day vs early night). *nvCiPC* showed a similar pattern of differential gene expression as *nvClock*, the protein that it regulates through phosphorylation in mammalian species [38]. *Timeout* (not shown in Fig. 6) is evolutionarily related to *Timeless*, a critical component of the *Drosophila* circadian clock, but the role, if any, of *Timeout* in cnidarian clocks is unknown. In our study, *nvTimeout* showed diel expression only under red light, and was up-regulated during the day. In the facultatively symbiotic sea anemone, *Exaiptasia diaphana*, *Timeless* expression (*Timeout* in other cnidarians, see Reitzel et al. [34]) was dependent on the presence or absence of symbionts and had a circatidal (12-h) rhythmic expression in the absence of *Symbiodinium* [39]. The overall gene expression results from our study indicate rich transcriptional responses to individual light colors, including differences in the expression of circadian clock components. Interestingly, patterns observed in blue and green light are highly similar, while expression in red light differed tremendously. One hypothesis for these shifts in global gene expression in red light is masking, where under typical daylight conditions, full spectrum illumination overrides or ‘masks’ other cues, eliminating the potential for differential behaviors in isolated spectra.

In coastal habitats, organisms experience complex environmental signals including solar, lunar, and tidal cues. To accommodate this, some marine invertebrates exhibit twice-daily oscillations in activity [40–42] and have even evolved separate circadian and circatidal oscillators [43–45]. Through locomotive tracking of individuals exposed to different color light treatments, we observed 24-h periodicity in sea anemones entrained to blue and green light:dark cycles; but surprisingly, we observed a 12-h activity rhythm in animals entrained to red light cycles and in constant darkness. While no evidence to date has supported the presence of a circatidal clock in cnidarians, Hendricks et al. [29] observed a similar twice-daily activity pattern in a portion of *Nematostella* that were LD entrained for 7 days, then moved to constant darkness and behaviorally monitored for 3 days. Additional behavioral data in *Nematostella* reports circadian behavioral oscillations persist for 2 days after being transferred from LD to DD conditions [46]. These studies suggest the presence of an endogenous oscillator that continues to keep a diel rhythm for at least 2 days, however the field lacks data on the behavior of anemones in prolonged darkness. The results presented here suggest that after 30 days in red light or in constant darkness, anemones do not show circadian behavior, but rather a bimodal activity pattern similar to circatidal rhythms of other estuarine invertebrates, pointing to the presence of a second oscillator within cnidarians, which may be masked by a predominate circadian clock. Deciphering the mechanisms of these two potential time keeping systems would be an impactful area of future investigation, providing a novel evolutionary perspective on cnidarian clocks. Considered alone, the similar behavioral profiles of animals entrained to red light and constant darkness might seem to suggest animals are not sensitive to red light and behave as if there is no light present; however, the vastly different gene expression profiles between red light and DD suggests the opposite.

A number of studies with cnidarians have shown diverse behavioral and reproductive responses to portions of the light spectrum including vertical migrations in the water column [15, 16, 47–49], phototaxis and photokinesis [50–53], gametogenesis [54, 55], tentacle expansion and contraction [23, 56–59], and feeding [60]. While sensitivity to blue and green wavelengths is common, spectral variation beyond this range (including red) has been suggested for some species of corals [61, 62] where spectral cues from both the water column and substrate influence larval positioning for settlement and metamorphosis [21, 63, 64]. Most of these studies have focused on ocular photoreception in species with dedicated visual structures, leaving relatively unknown the light-sensitivity and behavior of species with extraocular or non-visual photoreceptive mechanisms, particularly in anthozoan cnidarians (the class of cnidarians that includes anemones and corals; reviewed by Martin [65]). The results described here indicate that behavior and gene expression in the eyeless sea anemone *Nematostella* are dependent on the spectral range of light and the time-of-day, as is evident from differential organismal and molecular regulation. However, it is unknown what specific genomic mechanisms are used to interpret the dynamic light environment. Some studies have suggested opsin-mediated pathways are responsible for photobehaviors in other cnidarians like corals [66, 67], *Hydra* [24], and jellyfish [25, 26, 68], while cryptochromes are attributed to phototaxis by sponge larva [69] and the reproduction of some coral species [20, 70]. But few studies have functionally characterized the roles of these photopigments in cnidarians [66, 68, 71]. Ours and other previous transcriptomic data show that Type I cryptochromes (i.e., *nvCry1a*, *nvCry1b)* in *Nematostella* and corals are light-dependent with peak expression during the photoperiod, but lose rhythmicity in prolonged darkness (this study [20, 33–35, 70, 72–74];). Expression of Type I cryptochromes from corals, *AmCry1* and *FfCry1* (from *A. millepora* and *F. fragum,* respectively) are strongly diurnal in response to light cycles [20, 70, 73], particularly moonlight which is enriched in the blue wavelength range. A gene expression study by Reitzel et al. [34] suggested that different wavelengths of light may exert specific effects on *Nematostella*, specifically finding that Type I cryptochromes had differential expression dependent on which portion of the light spectrum (e.g., blue vs. longer wavelength) they were exposed to. There is variation with reports of Type II cryptochrome expression in *Nematostella*; for example, *nvCry2* does not show a strong diurnal response in any light treatment of this study as is similarly reported in Leach and Reitzel [33] and Reitzel et al. [34], which is unsurprising as this gene is most closely related to insect light-insensitive cryptochromes [75–79]. These data are in contrast to a study by Peres et al. [35], in which *nvCry2* expression displayed rhythmic oscillations in response to light:dark treatment. Furthermore, cryptochromes have been hypothesized to form feedback loops in the circadian circuitry of cnidarians acting as transcriptional repressors [13], although their spectral sensitivity has not been measured. In the only study to explicitly measure the absorptive range of a photoreceptor involved in cnidarian vision, Koyanagi et al. [68] found a green-light sensitive opsin is responsible for initiating the phototransduction cascade in box jellyfish. Screening of the *Nematostella* genome has identified 31 candidate opsins [26, 80], none of which have been functionally or spectrally tested. Of these, 11 transcripts are present in the Vienna transcriptome. Gene expression data from our study do not support rhythmic transcription of these putative opsin genes identified by Suga et al. [26] and transcript mining from the LD time course data set by Leach and Reitzel [33] did not reveal differential expression of any *Nematostella* opsins. Further, qPCR validation of select opsins did not show light-dependent expression (data not shown), but this result is not conclusive to exclude opsins’ role in the photobehavior of *Nematostella*. Although no study has examined the post-translational protein modifications in cnidarian circadian clock genes or photoreceptors, they are undoubtedly important. Multiple types of modifications to core circadian proteins and photoreceptors have been described for several species, including *Drosophila,* mammals, and cyanobacteria (reviewed in [81]). Future experiments are needed to determine the specific photoabsorptive properties of proteins and their post-translational modifications, like opsins, in species with extraocular vision to determine their non-visual functionality.

## Conclusions

The data presented here contributes to our knowledge of non-visual photobehavior and gene expression in an eyeless cnidarian from an integrative organismal and molecular context. The oscillating activity of *Nematostella* in red, green, and blue light:dark cycles strongly suggests differential mechanisms are responsible for photodetection of different light colors potentially due to a broader spectral sensitivity than previously thought. Further, our results indicate – for the first time in this species – red light sensitivity that is masked by light in the blue-green range along with a potential circatidal oscillator as evidenced by twice-daily behavioral rhythms in red light and in constant darkness.

## Methods

### Animal culture

Adult *Nematostella vectensis*, originally collected from Maryland and laboratory bred [82], were maintained in a laboratory setting as described in Hand & Uhlinger [83]. Sea anemones were kept in glass Pyrex dishes with 15 parts per thousand (ppt) artificial seawater (ASW). Individuals were fed haphazardly 3 days each week with freshly hatched brine shrimp (*Artemia sp.*) and the water was changed bi-weekly.

### Experimental treatments

Animals were split into four experimental light treatment groups and culture conditions were adjusted to simulate a diel light cycle (12-h light: 12-h dark; LD) or constant darkness (DD) inside a light- and temperature-controlled room. For 1 month, during the ‘entrainment period’, sea anemones were exposed to one of four isolated light treatments of different colors using Minger LED strip lights: (i) red LD, (ii) green LD, (iii) blue LD, and (iv) 24-h constant darkness, measured with a light meter (Fisher Scientific Traceable®) and adjusted for like intensities (red λ = 700; green λ = 560; blue λ = 450; range: 120–160 lx; Figure S1). For LD groups, light cycling began at 7:00 AM EST or Zeitgeber time (ZT) = 0 with “lights on”, and “lights off” at 7:00 PM EST or ZT = 12. During the entrainment period, animals were cultured on the same feeding and water change schedule as previous and care was taken for all groups during to limit stress to the animals. Animals in the DD group were fed and water changed during “lights off” to eliminate the potential for light contamination. All animals were starved for 2 days prior to data collection.

### Behavioral assays and data analysis

Noldus Ethovision (Noldus Information Technology) was used to record and quantify the movement of sea anemones in each light condition independently. An infrared 850 nm 5050 LED strip light (Environmental Lights) was used to facilitate recordings in both light and dark conditions. For each experiment, animals were measured in individual glass petri (9 cm across) dishes with 50 mL ASW (Figure S1). Video recordings were obtained for 12–16 animals in each light condition over 48 h (*n* = 60), beginning at ZT = 0. Animals were not fed during data collection, and recording time was minimized to reduce the impact of starvation on the measurements.

Each video recording was analyzed using Noldus Ethovision XT9 with the area of each petri dish set as the ‘tracking arena’. To avoid including light reflected off of the glass dish inside the tracking arena, all arenas were drawn with a 1 cm buffer from the edges of the dish (tracking arena area ≤ 50.27 cm^2^). In the case of animal movement into this region, the sample was discarded. Detection settings were set as follows: center-point detection, grey scaling (30–65), high pixel smoothing with contour erosion set to 1, and a sampling rate of 5.0 to ensure animal movement was detected throughout the collection period. Measurements of locomotion or ‘distance traveled’ in centimeters every 5 s was binned into hourly intervals (cm/hour) and analyzed with ClockLab software (Actimetrics).

Female populations were induced to spawn under red, green, and blue light cycles, following the animal care protocol outlined in Fritzenwanker & Technau [31]. Egg production was qualitatively recorded weekly to determine reproductive entrainment to individual light colors.

### Experimental sampling

For whole-organism gene expression analysis, animals were entrained in the same four experimental groups as described above for 1 month. After a starvation period of 2 days, individual sea anemones were sampled from each condition (4 biological replicates per time point) every 4 h over 24 h for a total of six timepoints (*n* = 96, replicates were not pooled), beginning at ZT = 2 (Figure S2). Samples were immediately preserved in RNAlater (Ambion) and stored at 4 °C until processing.

### Tag-based RNA library preparation, sequencing, and processing

Total RNA was isolated from 96 samples using the RNAqueous kit (Ambion) according to the manufacturer’s protocol. Briefly, after pipetting off and discarding RNAlater from each sample, whole animals were lysed by pipetting in lysis buffer for < 2 min, washed 2–3 times, and eluted on a column. Genomic DNA was removed using DNA-*free* kit (Invitrogen), and RNA was assessed using a NanoDrop 2000 spectrophotometer (Thermo Fisher Scientific). RNA was shipped for tag-based library preparation at the University of Texas at Austin’s Genomic Sequencing and Analysis Facility (GSAF) as in Meyer et al. [32] and adapted for Illumina HiSeq 2500. Briefly, total RNA was heat-fragmented and then reverse transcribed into first-strand cDNA. The cDNA was purified using AMPure beads, and PCR-amplified for 18 cycles. Unique Illumina barcodes were added in an additional PCR step for indexing of each sample. After an additional purification step, libraries were pooled, quality checked using a Bioanalyzer (Agilent), and size-selected using BluePippin (350-550 bp fragments). Raw sequence data from 100 base paired, single-end reads were delivered from the UT Austin GSAF. Raw reads were trimmed and quality-filtered using the FastX-toolkit [84]. Trimmed reads were mapped against the *Nematostella* Vienna transcriptome using the Bowtie2 aligner [85] and a read-counts-per-gene file was generated retaining only reads mapping to a single gene. Lastly, counts were imported into the R environment for all downstream statistical analysis (R3.5.0, R Core Team 2015). A full version of the library preparation protocol and associated bioinformatic tools can be found at https://github.com/z0on/tag-based_RNAseq.

### Gene expression analysis

Normalization and differential expression analysis of read counts was performed using the R package DESeq2 [86]. To enhance the rate of differential gene discovery, transcripts with low abundances (mean count < 3) were independently filtered as per the DESeq2 pipeline described in Love et al. [86]. The arrayQualityMetrics package [87] was used to detect outlier transcripts. DESeq2 normalized count data were regularized log transformed using the *rlog* function. Wald statistical tests were performed to identify diel transcription patterns in contrasts of all treatments and time points. *P*-values were Benjamini-Hochberg-adjusted to determine significance of contrasts (10% FDR cutoff). A rank-based gene ontology (GO) enrichment analysis was performed using signed, unadjusted log-transformed *p*-values (positive if up-regulated, negative if down-regulated) with the GO_MWU R package (https://github.com/z0on/GO_MWU) for all contrasts. We used the weighted correlation network analysis package in R to determine gene co-expression, using a soft threshold power of 11.5 [88]. Modules with expression patterns that were correlated greater than Pearson’s R > 0.45 were merged and GO enrichment analysis was performed using a Fisher’s exact test in the GO_MWU package. The R packages ggplot2 and pheatmap were used to generate graphs and heatmaps, respectively [89, 90].

## Supplementary information

**Additional file 1: Figure S1.** A. Light spectra intensity and energy values from the experimental treatments used in this study. Spectra were determined using a Qstick Subminiature Spectrometer (RGB Laser Systems). B. Energy values were determined using a radiometer (QSL 2100, Biospherical Instruments Inc.) at two positions in a 90 mm petri dish, on the inner (left star) and outer (right star) edges. Measurements are in μmol/cm^2^/sec of photons. **Figure S2.** Experimental design of *Nematostella* RNA sequencing experiment*.* Shown are the 12:12 h light:dark treatments of red-, green-, and blue-light treated and 12:12 h dark:dark treated anemones’ 24-h time-course experiment ran in parallel with each other. Solid colored red, green, blue, and grey boxes represent the ‘day’ period or photoperiod of 12 h, solid black boxes represent the ‘night’ period or scotoperiod of 12 h. The black, left pointing arrows represent the 30-day entrainment period prior to animal collections. The sampling points are shown in Zeitgeber Times (ZT) and as white circles. ZT = 0, or 0700, corresponds to “lights on” and ZT = 12, or 1900, corresponds to “lights off”. The sampling size (n) of each treatment is indicated on the left of each timeline. At each collection time point, which occurred every 4 h for 24 h beginning at ZT = 2, or 0900, 4 anemones per time point were sampled individually from each treatment (6 time points * 4 replicates = 24 anemones per treatment). A total of 96 individuals were collected during the 24-h time course. **Figure S3.** Weighted Gene Co-expression Network Analysis (WGCNA) and Module-Trait Relationships. Heatmap of transcripts (4965) assigned to 10 modules (arbitrary colors on the left of the heatmap). Eigengenes were calculated for each module. The strength of the correlations between traits (light treatment, time, biological replicates) and gene expression, is indicated by the intensity of the colored blocks with red and blue indicting positive and negative correlations. The numbers in each block represent the Pearson’s correlation between the module eigengene and the trait and corresponding *p*-values. Modules that are specifically correlated with each of the light conditions are marked with asterisks.

**Additional file 2: Table S1.** Photoperiod vs. scotoperiod statistical comparisons of activity profiles in this study. **Table S2.** Reads per sample reported in this study. **Table S3.** Differentially expressed genes by color and time from DESeq2 analyses. **Table S4.** Gene ontology enrichment for modules in gene co-expression analysis. **Table S5.** Gene expression comparisons for the candidate circadian genes reported in this study.

## Data Availability

The data discussed in this publication have been deposited in NCBI’s Gene Expression Omnibus (Leach and Reitzel, 2019) and are accessible through GEO Series accession number GSE132526 (https://www.ncbi.nlm.nih.gov/geo/query/acc.cgi?acc=GSE132526).

## Abbreviations

GO: Gene Ontology
LD: Light:dark
DD: Dark:dark
FDR: False discovery rate
ZT: Zeitgeber time
HSP: Heat shock protein
*helt*: Hairy and enhancer of split-related protein
*PAR-bZIP*: Proline and acidic amino acid-rich basic leucine zipper
CRY: Cryptochrome
*CiPC*: Circadian interacting pacemaker protein
qPCR: Quantitative polymerase chain reaction
PPT: Parts per thousand
ASW: Artificial seawater
EST: Eastern standard time
LED: Light-emitting diode
