# Supplementary figures and images for: Decoupling behavioral and transcriptional responses to color in an eyeless cnidarian
Source: BMC Genomics. 2020 May 14;21:361. doi: 10.1186/s12864-020-6766-y (PMC7222589; doi:10.1186/s12864-020-6766-y)

A.

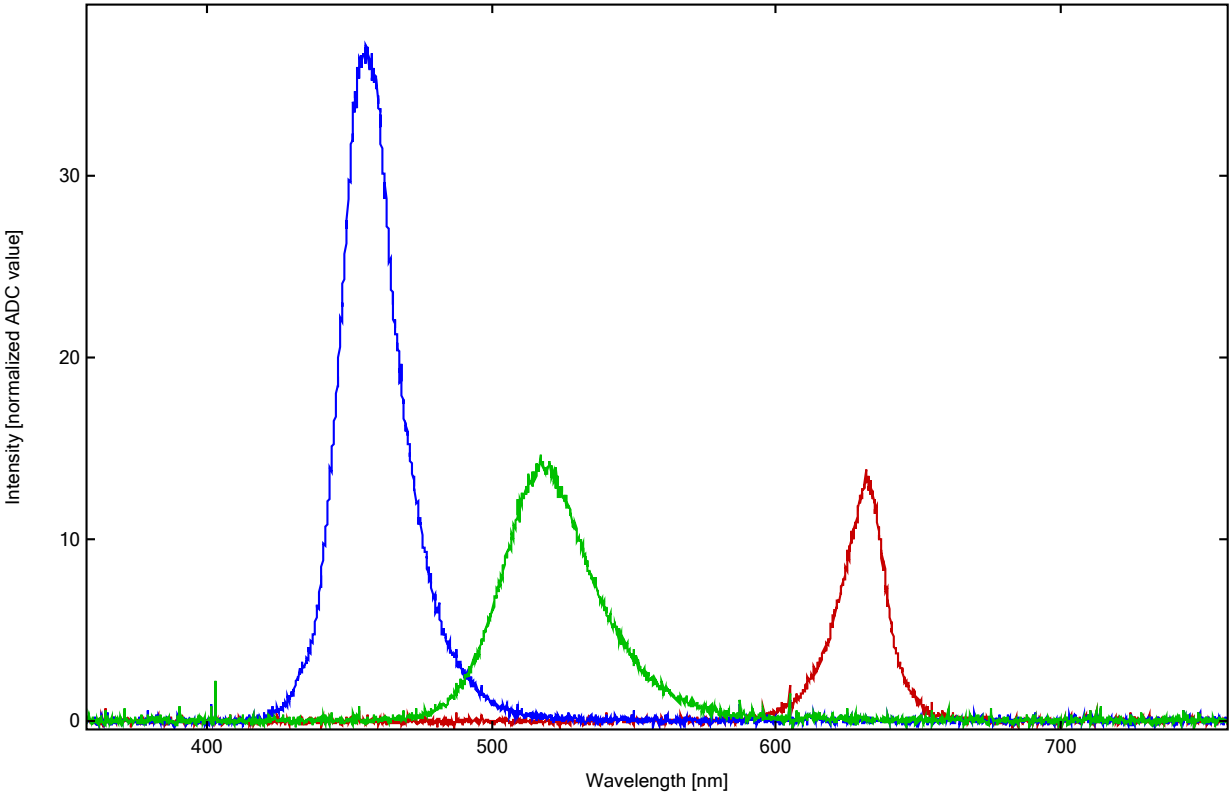

B.

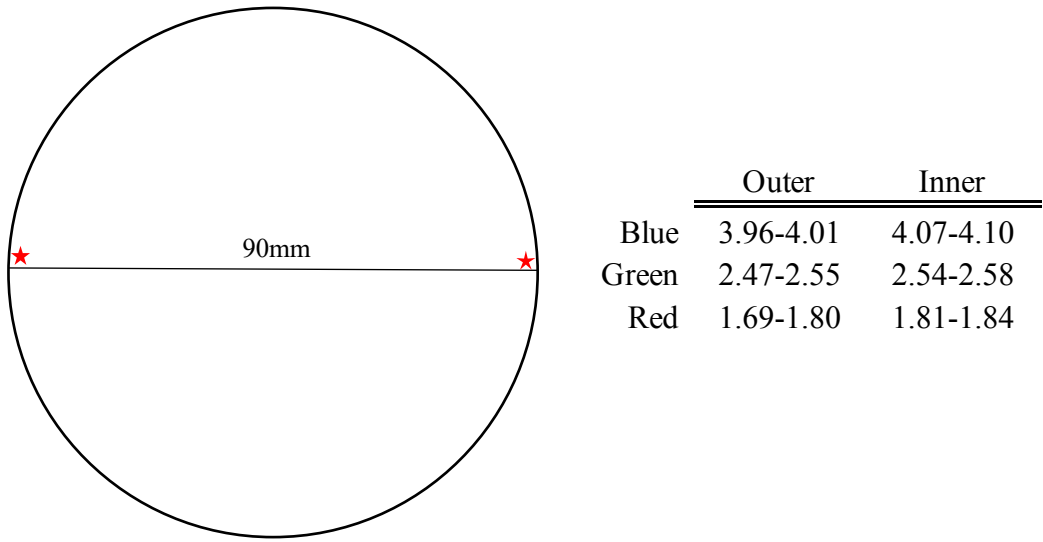

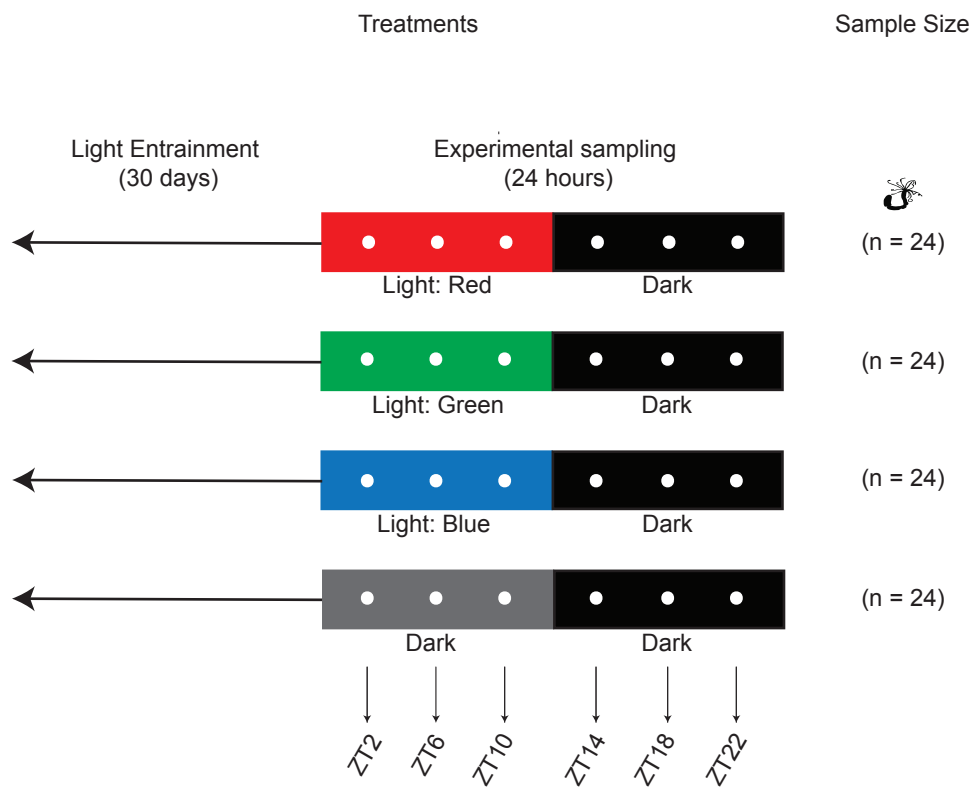

Module-trait relationships

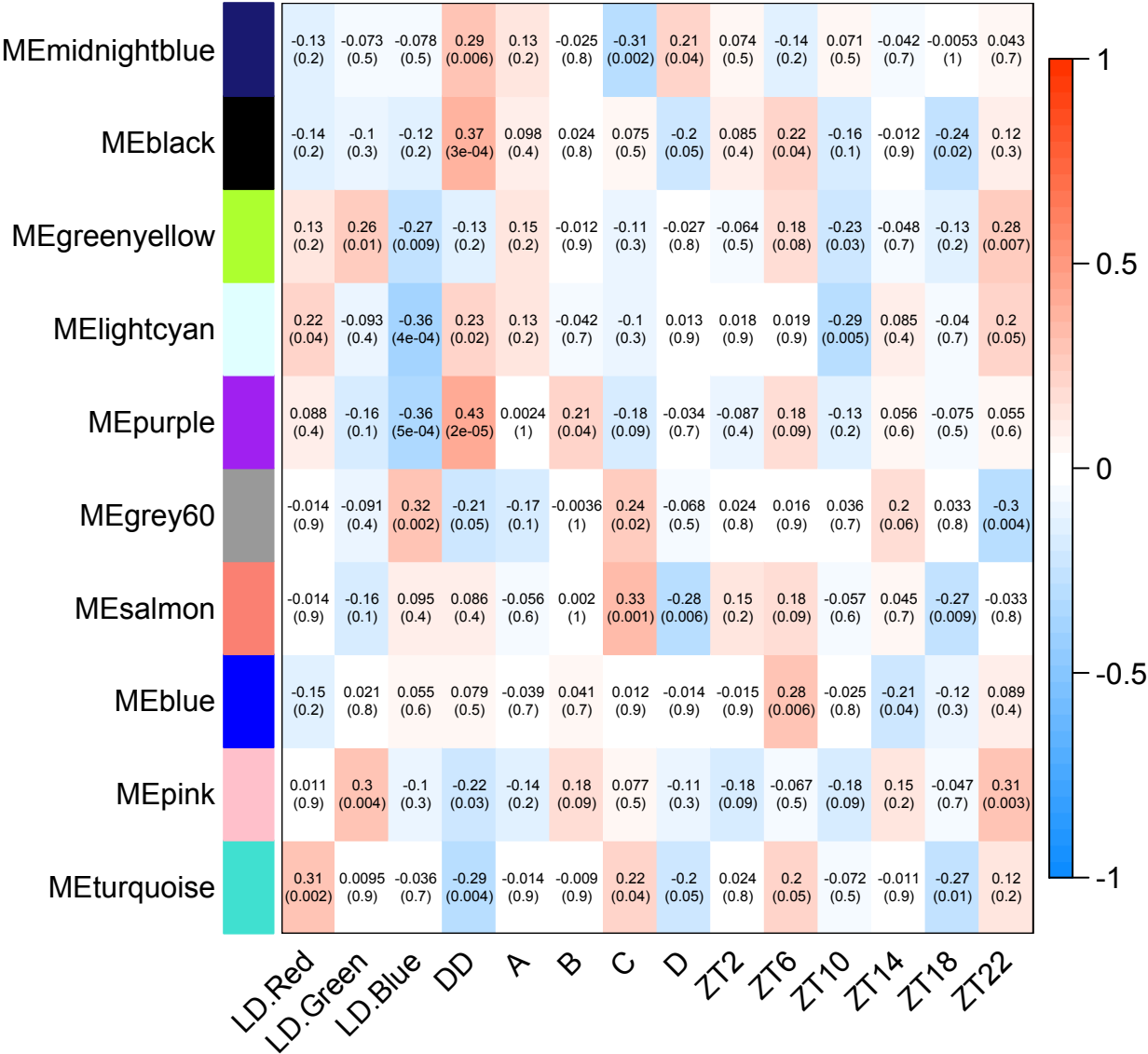

Supplement: Supplementary file 1 — Additional file 1: Figure S1. A. Light spectra intensity and energy values from the experimental treatments used in this study. Spectra were determined using a Qstick Subminiature Spectrometer (RGB Laser Systems). B. Energy values were determined using a radiometer (QSL 2100, Biospherical Instruments Inc.) at two positions in a 90 mm petri dish, on the inner (left star) and outer (right star) edges. Measurements are in μmol/cm2/sec of photons. Figure S2. Experimental design of Nematostella RNA sequencing experiment. Shown are the 12:12 h light:dark treatments of red-, green-, and blue-light treated and 12:12 h dark:dark treated anemones’ 24-h time-course experiment ran in parallel with each other. Solid colored red, green, blue, and grey boxes represent the ‘day’ period or photoperiod of 12 h, solid black boxes represent the ‘night’ period or scotoperiod of 12 h. The black, left pointing arrows represent the 30-day entrainment period prior to animal collections. The sampling points are shown in Zeitgeber Times (ZT) and as white circles. ZT = 0, or 0700, corresponds to “lights on” and ZT = 12, or 1900, corresponds to “lights off”. The sampling size (n) of each treatment is indicated on the left of each timeline. At each collection time point, which occurred every 4 h for 24 h beginning at ZT = 2, or 0900, 4 anemones per time point were sampled individually from each treatment (6 time points * 4 replicates = 24 anemones per treatment). A total of 96 individuals were collected during the 24-h time course. Figure S3. Weighted Gene Co-expression Network Analysis (WGCNA) and Module-Trait Relationships. Heatmap of transcripts (4965) assigned to 10 modules (arbitrary colors on the left of the heatmap). Eigengenes were calculated for each module. The strength of the correlations between traits (light treatment, time, biological replicates) and gene expression, is indicated by the intensity of the colored blocks with red and blue indicting positive and negative correlations. [file 12864_2020_6766_MOESM1_ESM.pdf]
